# How Proprioception Gives Rise to Self-Others-Knowledge

**DOI:** 10.3389/fpsyg.2021.575945

**Published:** 2021-05-24

**Authors:** Anita Pacholik-Żuromska

**Affiliations:** Nicolaus Copernicus University in Toruń, Toruń, Poland

**Keywords:** proprioception, multisensory integration, bodily self-consciousness, social perception, body-social problem

## Introduction

The already rich professional literature broadly informs about the role of the body in establishing the self-others distinction (Jeannerod, 2006; Iacoboni, 2009; Kyselo, 2015; Maister et al., 2015; Noel et al., 2017; Palmer and Tsakiris, 2018). The internal sense of ownership and sense of agency are the fundamentals of self-identification (Jeannerod, 2004; Blanke et al., 2015; Tsakiris, 2016; Braun et al., 2018). The lack of these two fundamentals (natural or artificially induced) also conveys important information, specifically that the action was executed by someone else (Iacoboni, 2009; Tsakiris, 2010). In my opinion, owing to its neural fundamentals, bodily self-consciousness (BSC) not only allows us to differentiate between self and others but also leads to the propositional knowledge of the subject and influences the social functioning of the subject (the self-others-knowledge—SOK). Assigning the important role of the body and multisensory integration in the self-others distinction is not new (Keromnes et al., 2019). However, what I add to this opinion is the recognition of the role of proprioception in shaping propositional SOK, i.e., shaping a specific type of metacognition.

## The Role of Proprioception for Bodily Self-Consciousness

The viewpoint presented in this article is in line with the enactivism, which is in turn deeply rooted in the phenomenological tradition (Maturana and Varela, 1998; Gallagher, 2006). According to this line what forms the mind is the body and its interaction with the environment (Wilson, 2002; Di Paolo and De Jaegher, 2012). The role of the body has been described particularly in relation to neural mechanisms underlying BSC—especially multisensory integration constituting proprioception (Blanke and Metzinger, 2009; Blanke et al., 2015; Limanowski, 2017). BSC manifested in the sense of ownership and sense of agency is therefore a psychological outcome of the synchronisation of sensorimotor information (Cf. Jeannerod, 2006; Blanke et al., 2015). However, given that BSC, built from different information including proprioceptive cues, closes the subject in the internal loop at the neuronal level of information processing, the problem arises of a link between self-awareness and awareness of others, i.e., how an individual subject becomes a social subject.

Proprioception can be defined in different ways. On the one hand, it is an unconscious registration in the central nervous system of one's own joint positions (Gallagher, 2006). The information coming from proprioceptors placed in the inner ear and muscles is analysed and integrated by the brain, which on this basis creates a body scheme (Cole, 1995; Gallagher and Cole, 1995). On the other hand, proprioception can be understood as non-conceptual bodily self-awareness (Bermúdez, 2005). In this case it is a kind of direct knowledge about the subject's basic experiences, which are the sense of the body's position and of being embedded in the world (Seth, 2015). Although such sensations are usually consciously inaccessible, they become apparent by paying attention to the subject's own movement, especially by performing certain kinds of action controlling whether the proprioception works properly. The basic multisensory information processing, when integrated, produces an experience being a self—an autonomous minimal selfhood (Blanke and Metzinger, 2009; Blanke et al., 2015).

In ecological psychology represented by Gibson (2002), proprioception is understood as the awareness of the perceiver regarding their existence in the environment, which accompanies the perception of the environment, hence the perception and proprioception come together. Having in mind Gibson's theory of ecological self, I think that the particular constitution of BSC is possible thanks to the sense of vision playing an important role in proprioception by increasing one's self-experience as an individual distinctive from the rest of the world. It is the vision which provides information via exteroception regarding which objects do and do not belong to the body (Gibson, 2002, p. 78). An example is the distinction between object motion and locomotion. In the first case it is perception which reveals the movement of an object in the static environment; in the second case, it is proprioception which informs that the observed movement is the activity of the organism's own body (Cf. Gibson, 2002, p. 78). To illustrate this, one can use the example of an illusion which occurs while sitting on the train; while waiting for its departure, we observe another stationary train through the window and, suddenly, we see windows passing and believe that our train has started to move. However, after a second, we realise that our train is still static and that another train is moving. The example of the illusion of the movement shows how strong the visual information about our position towards other bodies is. The role of vision in the formation of BSC is also featured in the conception of associative system learning, where the motor representation of one's own movements and the sensory representation of this movement connect with each other increasingly whilst self-observation (Heyes, 2016).

## Self-Other Knowledge

I claim that not only BSC but also the particular constitution of a SOK is possible thanks to the sense of vision playing an important role in proprioception by increasing self-experience as being an individual distinctive from the rest of the world (Cf. Jeannerod, 2004; Limanowski and Friston, 2019). Vision serves as the exteroception, i.e., the perception of external objects; proprioception, on the other hand, gives information about the body itself (Gibson, 2002, p. 78). A synchronisation of sensorimotor information coming from vision and proprioception results in representation of the acting body (Cf. Limanowski and Friston, 2019). The recognition whether it is one's own body which is acting is an ultimate factor influencing self-other distinction (Jeannerod, 2004) and establishing self-representation as distinctive from the representation of the others (Palmer and Tsakiris, 2018).

My thesis is that the bodily mechanisms of self-recognition not only lead to distinction me-others (bodily self-others knowledge—BSOK) but also contribute to the formation of propositional SOK (PSOK), i.e., that non-conceptual bodily representations are transformed into conceptual ones, by a recognition of a movement as an action. The meaning is namely an intention: a goal of the movement performed. In other words the ascription of a meaning to the observed movement rests on a connexion of a movement with a goal. I suppose that the recognition of a movement as intentional is the link connecting BSC with metacognition: SK and SOK. The ability to make a distinction between one's own action and the action of others underlies recognition of intentions and, i.e., understanding the actions of others by ascribing them the intention on the basis of the subject's own intentions and goals. As representations can be divided into sensorimotor (bodily) and cognitive (conceptual), the knowledge of others can be divided into sensorimotor and conceptual (Mul et al., 2019). Given that sensorimotor representations underlie the conceptual, BSOK underlies PSOK. In other words, the transition from BSOK to PSOK is the transformation of the sensorimotor representation into conceptual ones.

The claim that vision and proprioception build BSC and consequently SK and SOK is not beyond dispute. For example O'Regan and Noë (2001) trait vision as a way of acting, i.e., active exploration of the environment, which may be an argument against the importance of vision in constructing self-other boundaries. However, this discussion does not apply to the position presented here. I claim, namely, that the internal sense of ownership and sense of agency are the fundamentals of self-identification in BSC. In other words, in BSC resting on vision and proprioception an agent gains the information that it is their body rather than someone else's which is acting and, by using this acting body, they can achieve the intended goal. Thus, the BSC constituted in action provides important information for BSOK: self-others distinction by ascribing the action to oneself or to the other. BSOK underlies the higher-level cognition involving conceptual knowledge. The bodily experience provides a basis for the development of an intentional level of understanding, i.e., PSOK. But why do we need propositional knowledge about others at all if the differentiation has already been made at the bodily level of information processing? The answer is simple: to understand their intentions, to ascribe them mental states, and to interpret them as rational agents. For all this we need a conceptualisation.

## A Brief Discussion of Alternative Approaches

An interesting conception how the neuronal basis gives rise to SOK without the involvement of proprioception refers to the role of mirror neurons system (MNS) in cognition, which facilitates a distinction between an agent's own action and the action of the others. There are two interpretations of the role of mirror neurons in cognition. The first is broader and states that mirror neurons are involved in reading the intentions of others; moreover, the activation of mirror neurons in humans occurs more often than in primitives, which could be associated with a wider range of interpretation of actions although the effect would be the same: equally fast selection of the appropriate response (Rizzolatti et al., 1996; Iacoboni, 2009). Mirror neurons can distinguish the same movement, but are associated with different intentions (Fogassi et al., 2005).

If the conception of MNS is so useful in the explanation of SOK, why should we look for an alternative? First of all, it is not clear what MNS contributes to. The discussion around the interpretation of mirror neurons revolves around the question of whether MNS is involved only in understanding motor activities or in identifying the intentional states of others, i.e., SOK. The second interpretation of the role of MNS is, by definition, more sceptical and states that the activation of mirror neurons is used to read the sequence of activities that the body has to learn; in other words, mirror neurons are for learning rather than for understanding the intentions of others (Jeannerod, 1994). To compromise rather than fully excluding MNS from the conception proposed in this article it is legitimate to say the reference to the multisensory integration as the neural basis for SOK incorporates MNS. The conception of MNS also contains elements such as movement, action recognition and ascription, the sense of ownership, and the sense of agency.

Another conception regarding how PSOK is created, refers to the ability of perspective taking, i.e., the ability of putting oneself in place of somebody else (Jeannerod, 2006). This conception is based on the Theory of Mind (ToM), which refers to the classical representation-based approach to social cognition where the ability to interact with others rests on the mental constituents of cognition rather than their physical counterparts (Tomasello and Rakoczy, 2003; Tomasello, 2019). Nonetheless, I believe that ToM is good for an explanation of the metacognition, i.e., it explains the mind-social problem but not the body-social problem (Kyselo, 2015). Research on disorders such as Autism and Schizophrenia, where agents have difficulties in interpersonal relations, show that problems with interaction abilities begin at the level of BSC formation, i.e., at the level of multisensory integration. In other words, the problem in SOK starts with the problem with bodily representation of self; specifically, in Autism a subject possesses a sharper self-others boundary which extends beyond the norm whereas in Schizophrenia this boundary is weaker (Noel et al., 2017). The abnormal size of this boundary is determined by disturbances in the processing of information about peripersonal space (Noel et al., 2017). The problems with mindreading or intentions understanding are a consequence of the impaired somatosensory representation of the self.

## Conclusion

**Figure 1:**
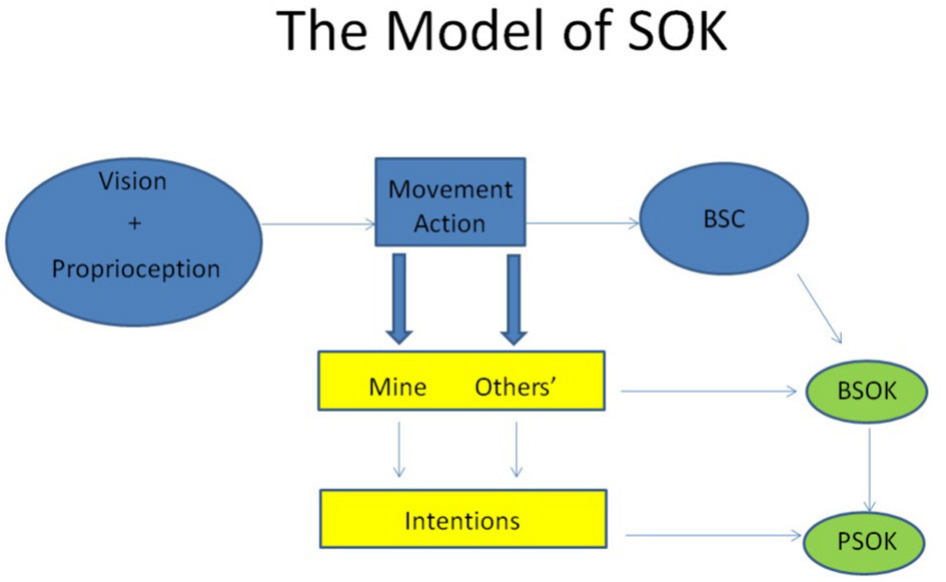
The model of SOK.

In my opinion the conceptualised knowledge about the others (PSOK), i.e., the recognition of other bodies as intentionally acting individuals necessarily involves the primacy of the non-conceptual bodily self-other knowledge (BSOK). Hence if one wants to build a model of how SOK is created, must refer to the neural constitution of BSC involving the sense of vision and proprioception, triggered and tuned in a goal-directed movement, i.e., an action (Figure 1). Vision and proprioception are namely significant indicators of the source of the action—the sense of agency. In such an account not only the individual mind, but also the social mind is shaped by body and its interaction with the world.

## Author Contributions

The author confirms being the sole contributor of this work and has approved it for publication.

## Conflict of Interest

The author declares that the research was conducted in the absence of any commercial or financial relationships that could be construed as a potential conflict of interest.
